# Smartphone fundus photography: a narrative review

**DOI:** 10.1186/s40942-021-00313-9

**Published:** 2021-06-08

**Authors:** Usama Iqbal

**Affiliations:** Department of Ophthalmology, Gujranwala Medical College/ DHQ Teaching Hospital , Gujranwala, Punjab Pakistan

**Keywords:** Fundus, Imaging, Smartphone, Retinal, Photography

## Abstract

**Background:**

The idea to use smartphone for fundus photography was put forward in 2010. Over the last decade, there has been a dramatic development in this field. This narrative review focuses on the principle of smartphone fundus photography, how to master this technique, problems encountered by the beginners, camera applications/devices designed for this purpose and the safety profile of smartphone flashlights for retinal photoreceptors.

**Discussion:**

Smartphone fundus photography using a condensing lens is based on the same principle as indirect ophthalmoscopy. Smartphone flashlight serves the purpose of light source or illuminating system. Real and inverted image of the retina is focused by the smartphone camera after adjustment of the filming distance. Beginners can face difficulties like adjustment of the filming distance, glare from condensing lens and reflection from the ceiling lights. Mobile camera applications and holding devices designed for this purpose can help the beginners to address these difficulties. There have been safety concerns about photo-biological risk for retinal photoreceptors by flashlight. Although the spectral irradiance on the retina, while using smartphone for fundus imaging is within the safety limits set by ISO 15004-2.2. The safety profile of latest model flashlights which deliver more power compared to older flashlights, need to be assessed.

**Conclusion:**

Smartphone fundus photography is a cheap, cost effective, portable and a convenient method for retinal imaging. With practice and use of smartphone camera applications designed for this purpose, the beginners can master this technique. By training young ophthalmology residents and ophthalmic primary caretakers, this retinal imaging technique can be utilized for artificial intelligence, patient diagnostic and educational purposes.

## History of retinal imaging

Historically the art to image the inner layer of the eye i.e., the retina has been of great interest for ophthalmologists. Over time, different techniques have been developed for imaging and analyzing the pictures of the retina. Originally, the retina has no color and is transparent. With proper techniques, it is possible to image the retina non-invasively [1]. The main problem encountered while retinal imaging is the optical system of the eye itself. Optics of the eye which results in the formation of an image of the surrounding objects on the retina hinders the direct inspection of the retina itself.

The idea to image the retina was put forward by a French physician Jean Mery. He demonstrated this in cats, that the retinal vessels become visible if a cat is submerged in water and pupils are observed from outside [1]. The first picture of the retina, showing the retinal vessels and gross anatomy was obtained by German Ophthalmologists Gerl in 1891 [2]. He later received Nobel Prize for his contribution. The first fundus camera was developed in the nineteenth century by Gullstrand [3]. The modern-day fundus camera is based on his model.

## Importance of retinal imaging in clinical practice

There has been a dramatic development in the field of retinal imaging over the last few years. Retinal vasculatures are the only part of the human body circulatory system which can be visualized non-invasively [4]. Fundus photographs can be helpful for documenting retinal as well as systemic diseases. These photographs can be subject to digital analysis for better understanding of micro-vascular complications of systemic diseases.

Smart phone based fundus photography is a convenient method because of its portability and cost effectiveness. It can be utilized for screening purposes, for early detection and to note the progression of a range of retinal pathologies including diabetic retinopathy, age related macular degeneration, retinal tumors, retinoblastoma, glaucoma and the list goes on.

Ramachandran et al. assessed and validated the digital analysis of smartphone fundus photographs using artificial intelligence based automated software [5]. They concluded that this method has a very high sensitivity for early detection of diabetic retinopathy and sight threatening diabetic eye disease. Tapan et al. utilized this idea for taking fundus photographs using smartphone in pediatric age group [6].

Recent advancements in the electronic media also increases the importance of photographs, which can be utilized as an aid in disease diagnosis introducing the concept of ‘tele ophthalmology’ [4]. Using this concept the primary care physicians, can take the pictures of fundus pathologies at their place of practice. These pictures can then be shared subject specialists to have their expert opinion. This practice reduces the need for direct referral of patient to tertiary care units for ‘in person’ examination.

## Retinal imaging systems

- Fundus cameras (mydriatic and non-mydriatic): The standard method for retinal imaging is by using a fundus camera. Fundus camera can be mydriatic, which requires pupil dilation and uses bright light flash, or non-mydriatic which is more advanced does not require pupil dilation and uses a flashlight of low intensity [7].
- Handheld and instrument mounted cameras: Hand-held fundus camera (based on the principle of traditional fundus camera), indirect ophthalmoscope mounted fundus camera, and slit-lamp mounted camera are also used for retinal imaging. These devices are mostly high cost and are used limitedly in the routine practice of ophthalmology [8].
- Use of smartphones for retinal imaging: High-quality cameras and high-resolution video features of smartphones can also be utilized for retina imaging. Lord et al. in 2010, published the first report on the use of smartphones for retinal imaging [9]. After this initial breakthrough, there has been much advancement in the field of smartphone-based retinal imaging. Initially, smartphone fundus photography was performed by hanging it to the eyepiece of the slit lamp and focusing the retina using a 78D or 90D condensing lens. This method required a great deal of hand stability and was difficult [10].

## Use of smartphone-based fundus photography in artificial intelligence

Rajalakshmi et al. evaluated the use of artificial intelligence-based interpretation of the fundus pictures captured by using a smartphone. They reported high sensitivity for this method in the detection of diabetic retinopathy, retinal detachment, and other retinal pathologies in patients presenting at a general practitioner clinic [5]. Training the primary health care providers working in basic health care facilities, to use smartphones for taking fundus pictures, can serve as a promising tool for screening purposes and avoiding unnecessary referrals to tertiary care hospitals.

## Principle of smartphone fundus photography using 20D lens

**Fig. 1:**
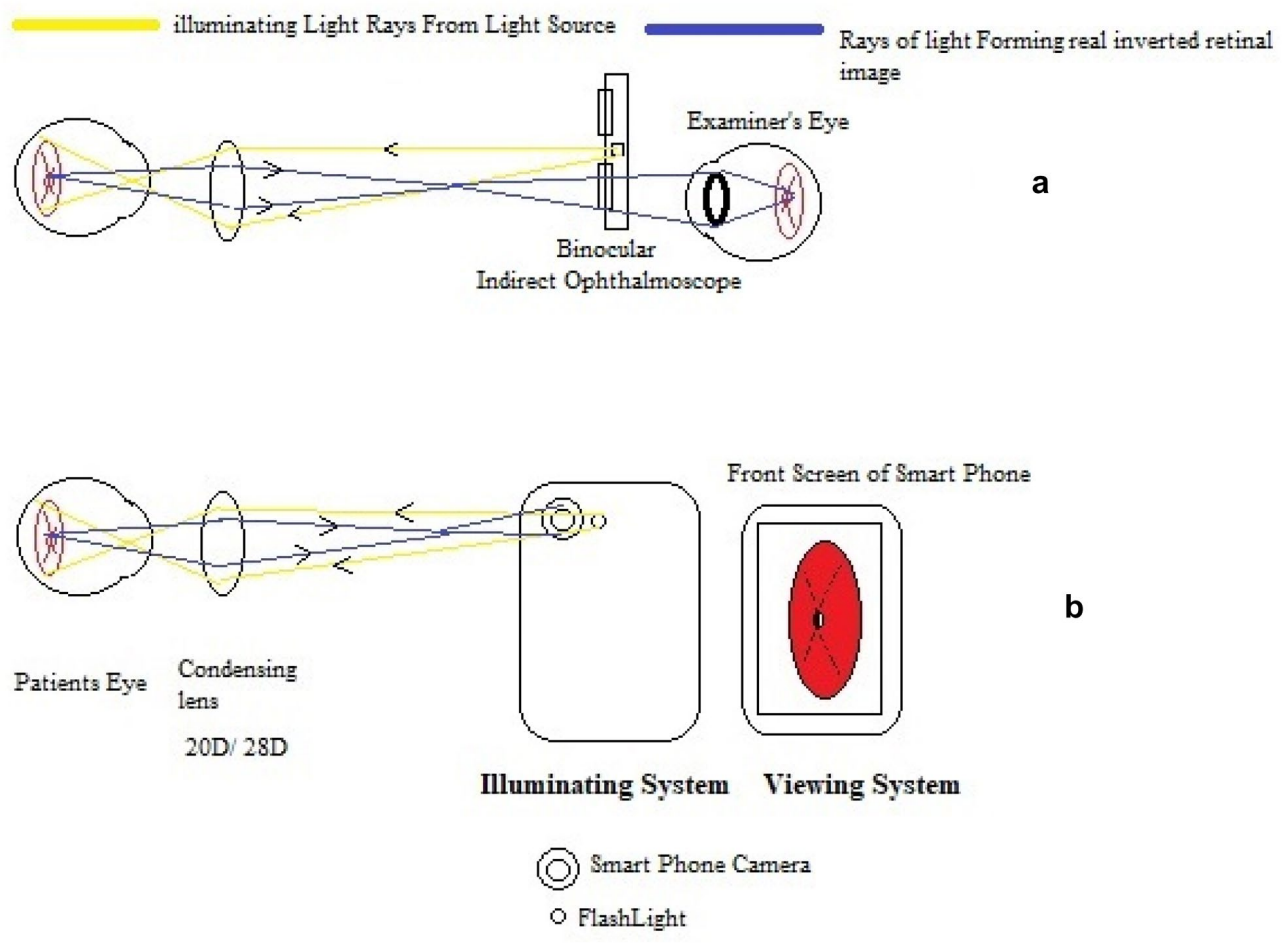
Comparison of the optical principle of indirect ophthalmoscopy and smartphone fundus photography. **A** Light source and viewing system of indirect ophthalmoscopy. **B** Light source and viewing system of Smartphone Fundus photography

Smartphone fundus photography using a 20D condensing lens is based on the same principle as indirect ophthalmoscopy. The viewer’s eye is replaced by the display screen of a smartphone. The light source of indirect ophthalmoscope is replaced by the flashlight of a smartphone camera (Fig. 1).

## Technique and positioning

After pupil dilatation, the smartphone camera application is opened. The camera mode is changed to video. The flashlight is turned on. In most smartphones (iPhone™, Samsung™, Huawei™) the flashlight remains continuously on when the camera is switched to video mode. 20D condensing lens is used to focus the fundus, while an image of the focused fundus becomes visible on the smartphone display screen.

**Fig. 2:**
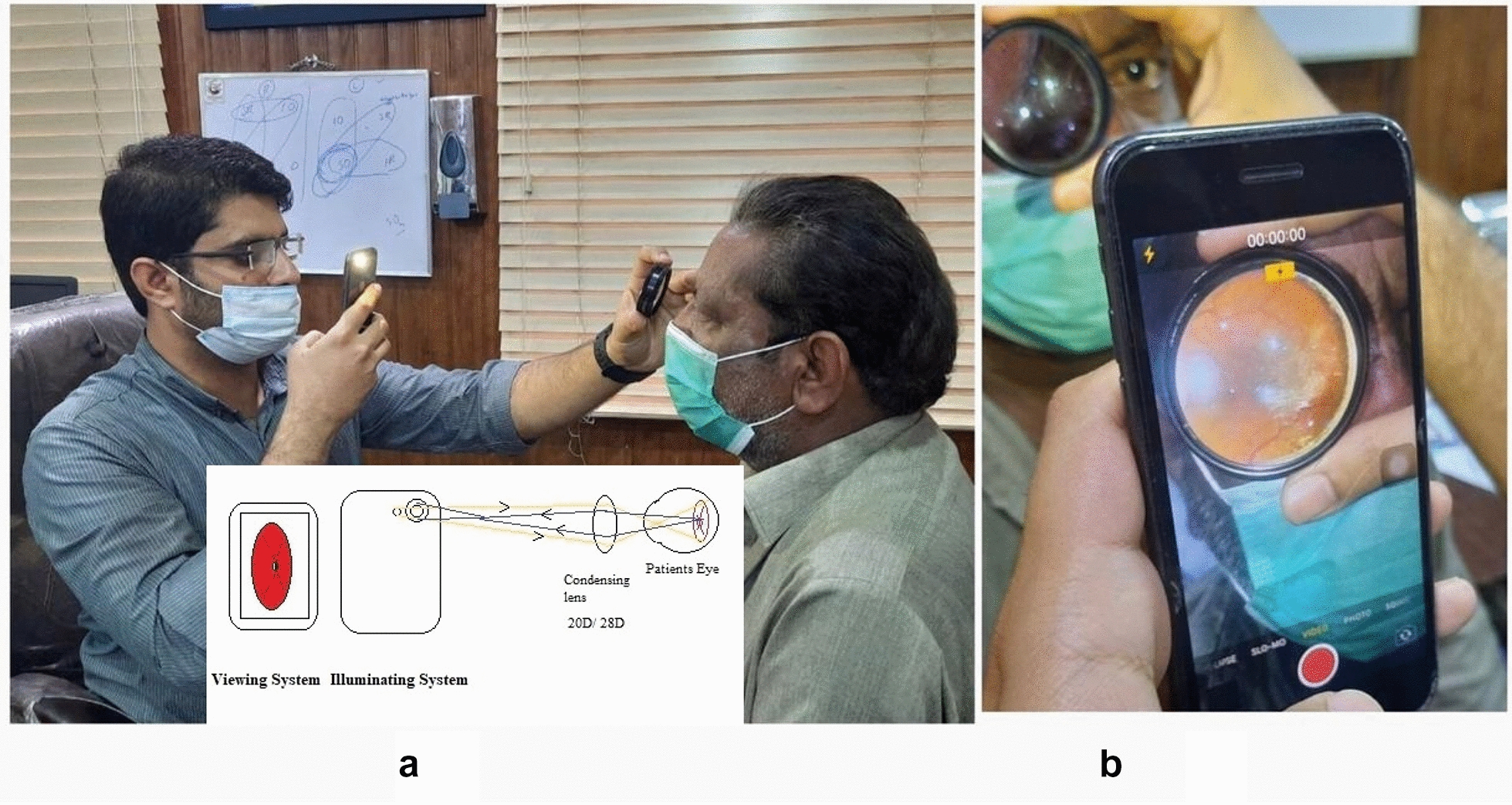
**A** Positioning of examiner with 20D lens in one hand and smartphone with a flashlight on in another hand. **B** View of the focused retina on the display screen of a smartphone

The positioning of the examiner and the patient are shown in Fig. 2.

Examiner holds the smartphone in one hand while the 20D lens is held in another hand near the patient’s eye. Examiner holds the lens between the thumb and index finger. The middle finger is used to elevate the eyelid while the little finger and ring finger stabilizes the hand on the patient’s forehead. The filming distance is adjusted by the forward or backward movement of the lens or the smartphone, to the point the fundus is focused and fills the full area of condensing lens on the smartphone display screen.

The technique to master is the adjustment of filming distance to the point at which fundus is focused. The picture of the fundus when focused and seen on a smartphone screen is then captured. The image of the retina is laterally inverted and upside down.

The magnified view of a retinal lesion is possible by moving the smartphone towards the patient. This is combined with the relative movement of the 20D lens away from the patient. In the same manner, to have a large field of view, the smartphone is held at a greater distance from the patient. By adjusting the patient’s gaze, different quadrants of the retina can be seen in the same manner as in indirect ophthalmoscopy.

## Features of smartphone which affect fundus focusing

The quality of image and ease of focusing the retina while adjusting the filming distance depends on these factors:

- Location of the camera lens on the backside of the smartphone;
- Location of a flashlight in relation to the camera lens.

Mostly the camera lens is located on the corner of the phone and the display of the fundus image is on the center of the phone. This difference in location of the camera lens and display screen requires maneuvers to focus the fundus image in the center of the display screen [11].

Regarding the location of a flashlight in relation to the camera lens, the closer the flashlight is to the camera lens, the easier it becomes to focus the retina.

## Difficulties faced by the beginners

The beginners can face a few problems at the start, while they are learning to adjust the filming distance. One difficulty highlighted by Raju et al. is that the phone flashlight is not continuously on if the phone is in picture mode [12]. Also in picture mode, it often becomes difficult to capture the image in a few seconds the fundus is in focus. The author recommends that if the mode of the phone camera is switched to video mode and the flashlight is turned on then it will remain on continuously. The examiner can focus the fundus and while it is focused the video shoot is turned on to film the video for the time the retina remains focused. The best screen from the video can then be saved by taking a screenshot. The problem highlighted with this method of video mode is that the resolution of an image in video mode is not as high as taken in still photography mode [12]. The use of the latest model smartphone with high-resolution video features and screen tap to focus the area of interest can address this problem of image quality.

**Fig. 3:**
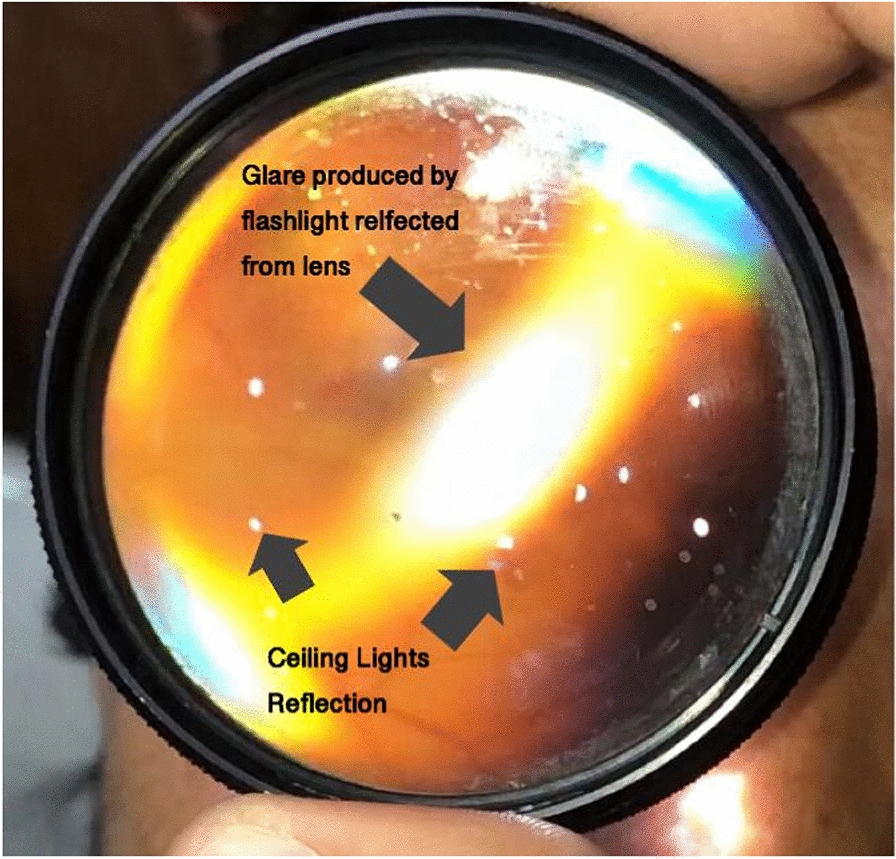
Flashlight glare and Reflection of examination room ceiling lights

Another problem faced by beginners is the glare caused by the reflection of a smartphone flashlight from a 20D lens. Also, the ceiling lights in the examination room can be reflected in the image (Fig. 3).

The glare due to the flashlight can be managed by a slight tilting maneuver of the examiner's hand holding the lens and notice the position of the lens where glare is least present. The reflection from examination room ceiling lights can be avoided by performing the procedure in a dark room.

The latest smartphones come with more than one camera lenses, it is required to note the camera lens which shoots the video, and then keeping in mind its position, the filming distance can be adjusted.

## Using different smartphones

Most of the available reports on fundus imaging using smartphones describe the technique using iPhone™. Authors have also shared their experience using Samsung™ S-series and Note series to capture the fundus images [12]. Other devices e.g., Huawei™, Oppo™, Vivo™ can also be used for this purpose. The examiner can get an idea about the filming distance by adjusting the relative position of the smartphone in relation to the lens. As per the author’s personal experience, devices in which the flashlight is close to the lens-camera are easier to be used for this purpose.

Raju et al. recommended that the use of a 28D lens makes it easier to focus the retina [12]. Using the feature of screen touch to focus the area of interest can also improve the image quality and details of the picture.

## Holding devices

To address the problem of filming distance adjustment, different devices are available which are attached to the smartphone and have a compartment to hold the 20D lens at a measured distance, co-axial with the camera lens [13]. The problem with these devices is that most of them are over-priced.

## Smartphone camera applications (app)

Smartphone applications that provide options for camera setting adjustment e.g., exposure setting, light intensity adjustment, manual focus option, and zoom setting are available for android OS, iOS (Apple Inc, Cupertino, USA)™, Windows mobile, Symbian, and Java operating systems. Names of a few of these applications are EyeTakes (for iOS), Shot Control (Android), ProCamera (iOS only), FiLMiC (Android and iOS), Camera FV-5, ProCapture, and Ullman indirect app [13]. Ullman indirect app allows the user to manually control the brightness of smartphone flashlight, flip fundus image to correct orientation, and import images with the need to screenshot from the video clip [14].

## Comparison with fundus camera pictures

Adam et al. conducted a trial for the reliability of smartphone-based retinal imaging in comparison with a standard fundus camera. They concluded that both these methods of retinal imaging are comparable in their diagnostic utility. The quality of fundus images taken by using a smartphone is also comparable with the standard fundus camera pictures [15].

## New devices

i-Examiner manufactured by (Welch Allyn, Skaneateles Falls, NY), is FDA approved to capture retinal images. It is used in combination with PanOptic Ophthalmoscope and its compatible mobile application. This device can capture the retinal image from an undilated pupil. Its field of view is 25° [16].

Russo et al. reported a device named D-Eye adapter (Si14 S.p.A., Padua, Italy). This device is attached magnetically to the smartphone. Its field of view is 200 degrees [16]. The retinal images taken with the help of a D-eye adapter are comparable to slit-lamp fundus images when examined by masked examiners, as reported by Russo et al. in their comparative study [16].

Other popular smartphone-based fundus photography gadgets available in the market include Peek Retina and Volk iNview™ [17]. Peek retina contains a clip that is attached to a smartphone. Volk iNview™ works with its compatible mobile application. This application provides additional features including data-keeping and patient records.

## Safety profile of smartphone flashlight for retina

The flashlight of smartphones, which is used as a light source in smartphone-based retinal imaging systems, may be a risk factor for photobiological damage to the retinal photoreceptors [18]. Different types of flashlights which are used in smartphone cameras include single light-emitting diode (LED), dual-LED flash, and xenon flash. Among these, single and dual LED flashlights are more commonly used by smartphone manufacturers while xenon flash is used with professional cameras. The radiant power of xenon flash is greater when compared with single or dual-LED flash [18]. The flashlights of smartphones are safe for their use while still photography and videography. When used for the purpose of fundus imaging, the exposure time to retinal photoreceptors exceeds the time while the flashlight is being used for videography or still photography.

International Organization for Standardization (ISO 15004-2.2) sets the safety limits profile for ophthalmic instruments. According to ISO 15004-2.2, spectral irradiance (W/cm^2^/nm) on the retina is calculated separately for thermal and photochemical action. Kim et al. demonstrated that for 1-min retinal exposure to smartphone flashlight, the retinal irradiance was 150 times below the limit for thermal damage, and 240 times below the limit for photochemical action [19].

Hong et al. also reported that photobiological risk by iPhone-based retinal imaging system was 1 order of magnitude below the safety limits set by ISO 15004-2.2 [20].

The latest models of smartphones developed recently can deliver 20 times more power compared to older flashlights. They can therefore pose an increased risk for biological damage to the retina [19]. The safety profile of these new flashlights should be assessed according to ISO 15004-2.2 before they are used for fundus imaging.

In summary, smartphone based fundus photography is a cheap, portable and a convenient method to image the retina. It can serve as an alternate to costly and high built conventional fundus cameras. The basic optical principle of smartphone fundus photography is that of indirect ophthalmoscopy. Ophthalmologists and young residents should master this art, which can be utilized for disease screening, educational and tele-medicine purposes. Beginners can face difficulties including adjustment of the filming distance, glare from condensing lens, ceiling lights reflection and adjusting the relative position of condensing lens in relation to the smartphone. With practice and noting the hand maneuvers while using your smartphone, these issues can be mastered. Holding devices and smartphone camera applications can also serve to address these issues. The risk of photo biological damage to retina by smartphone flashlight is very less if exposure time is about 1 min. The latest flashlights which are being developed by smartphone companies can deliver more power as compared to older flashlights. So their risk should be investigated before their use for fundus imaging.

## Figures and Tables

**Fig. 4:**
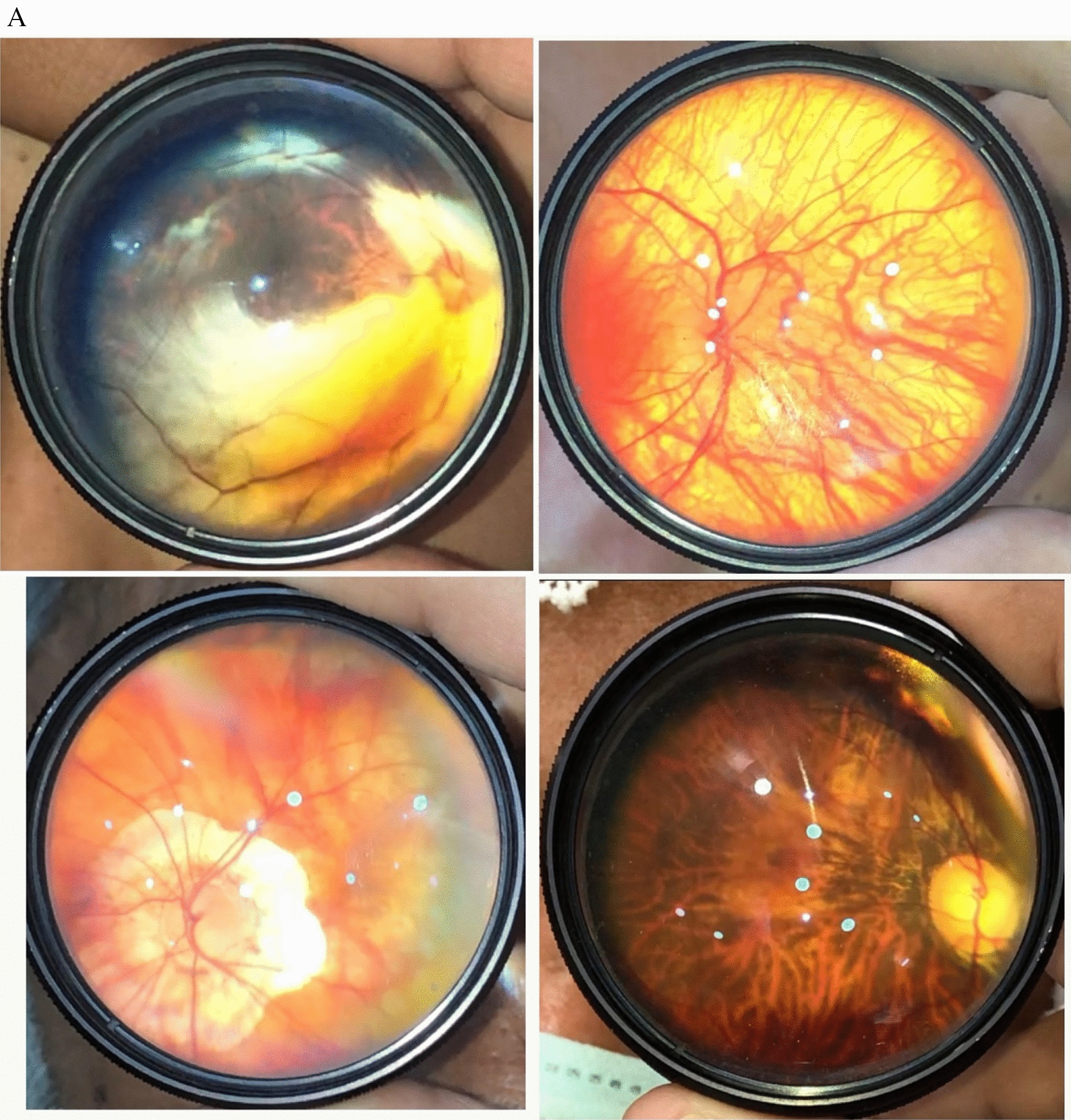
Hereditary retinal pathologies

**Fig. 5:**
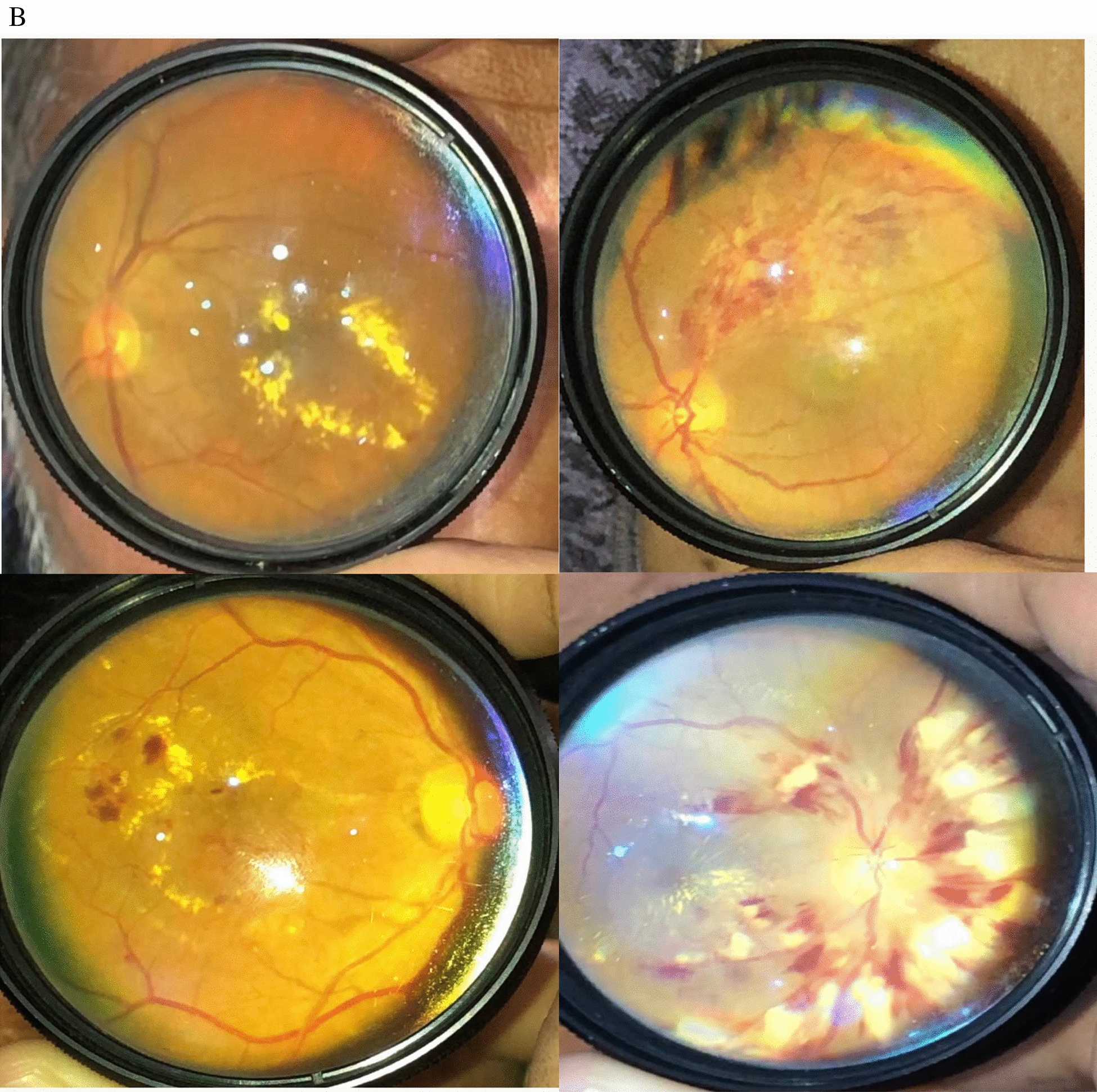
Diseases related to medical retina

**Fig. 6:**
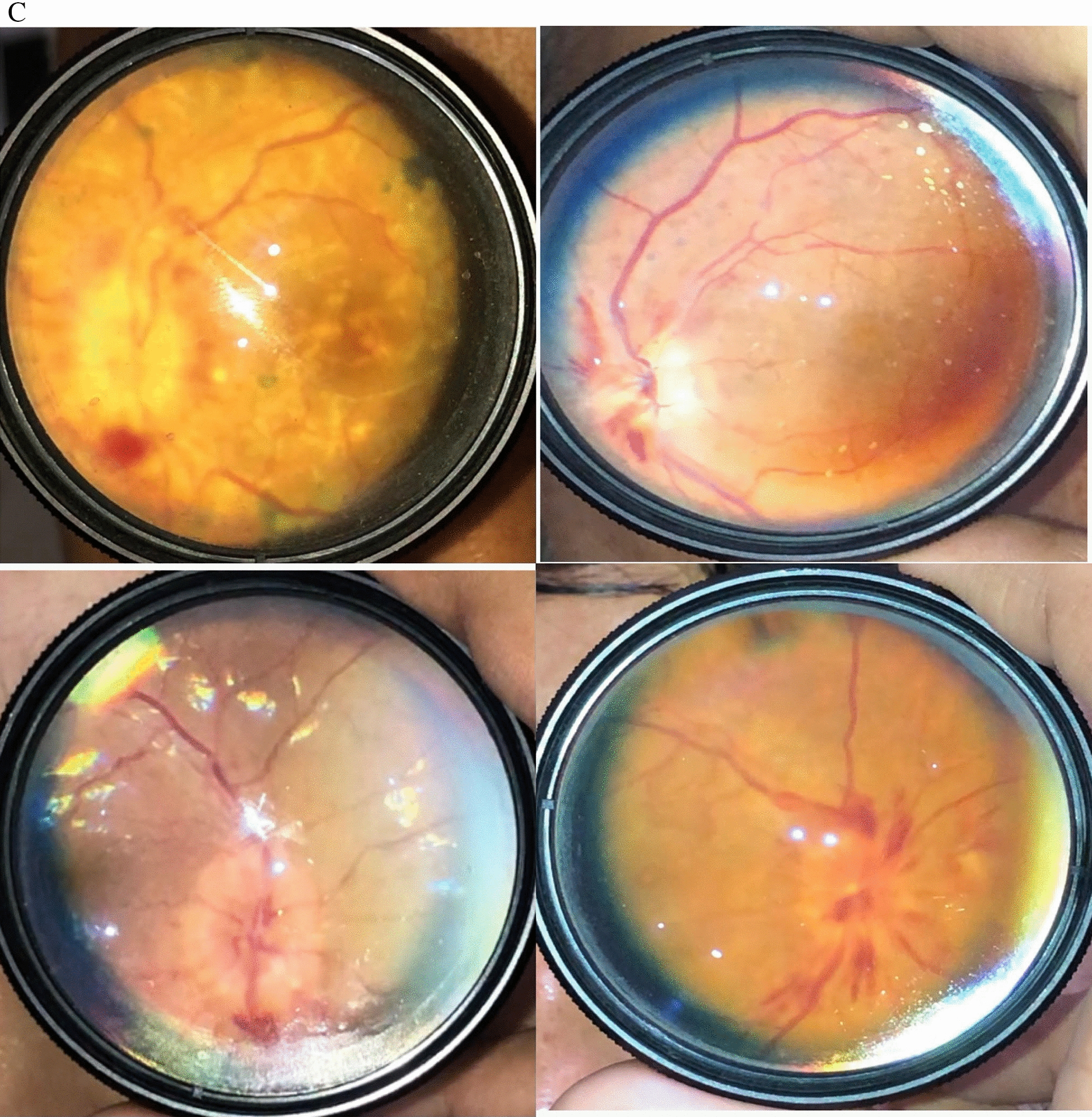
Fundus pictures showing different stages of optic disc swelling

**Fig. 7:**
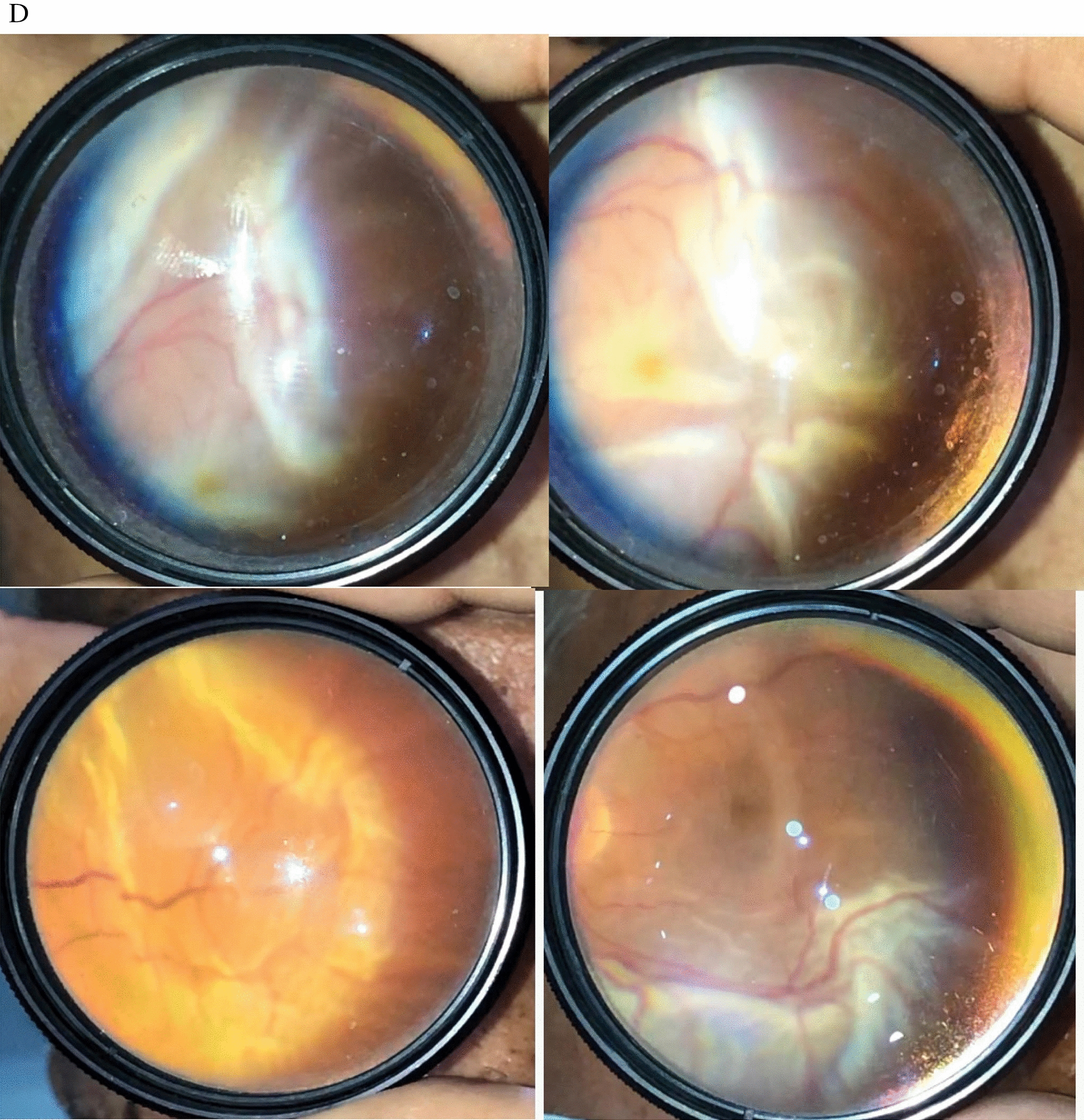
Fundus Pictures showing retinal detachment

**Fig. 8:**
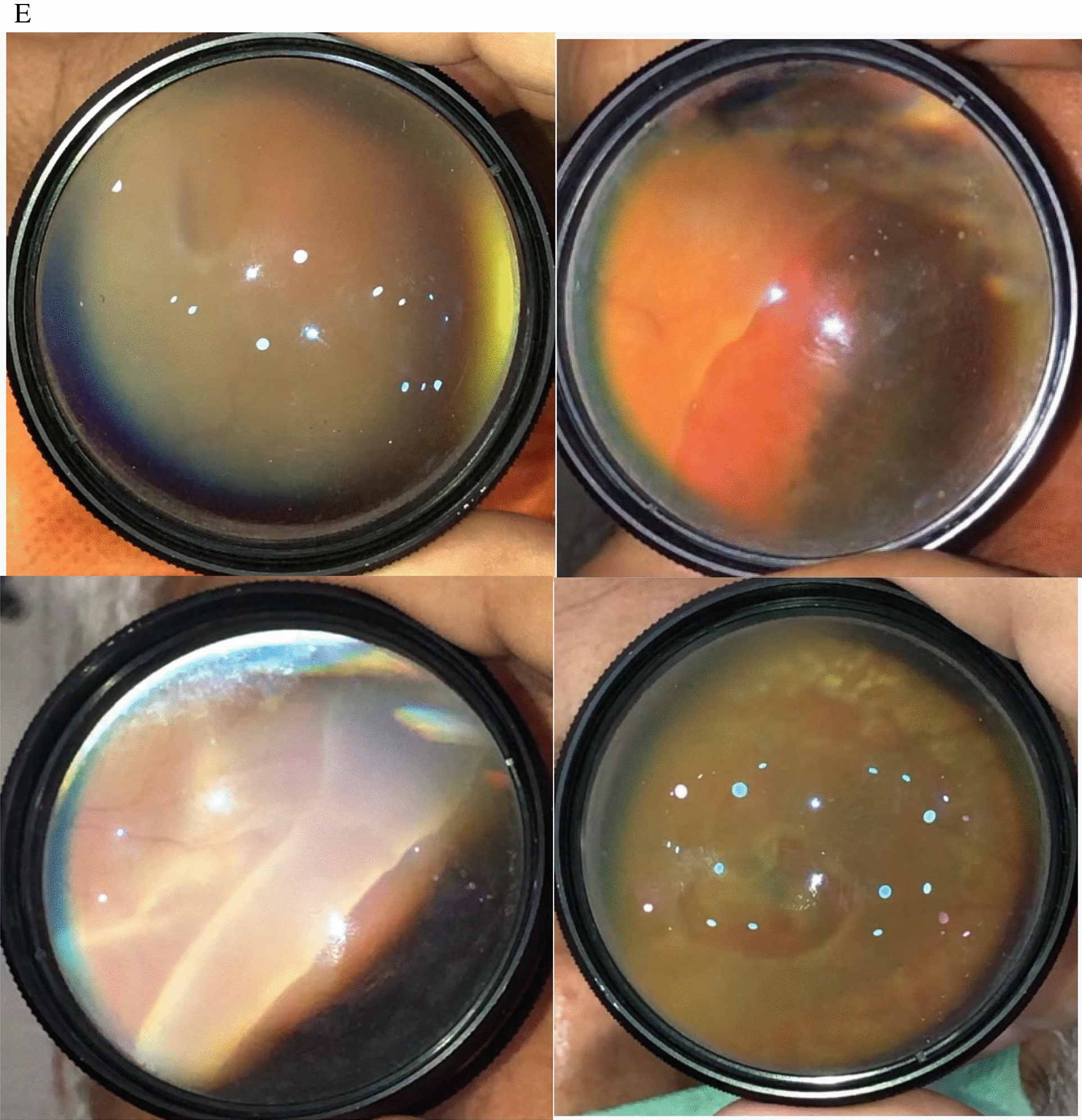
Fundus pictures showing different retinal breaks

**Fig. 9:**
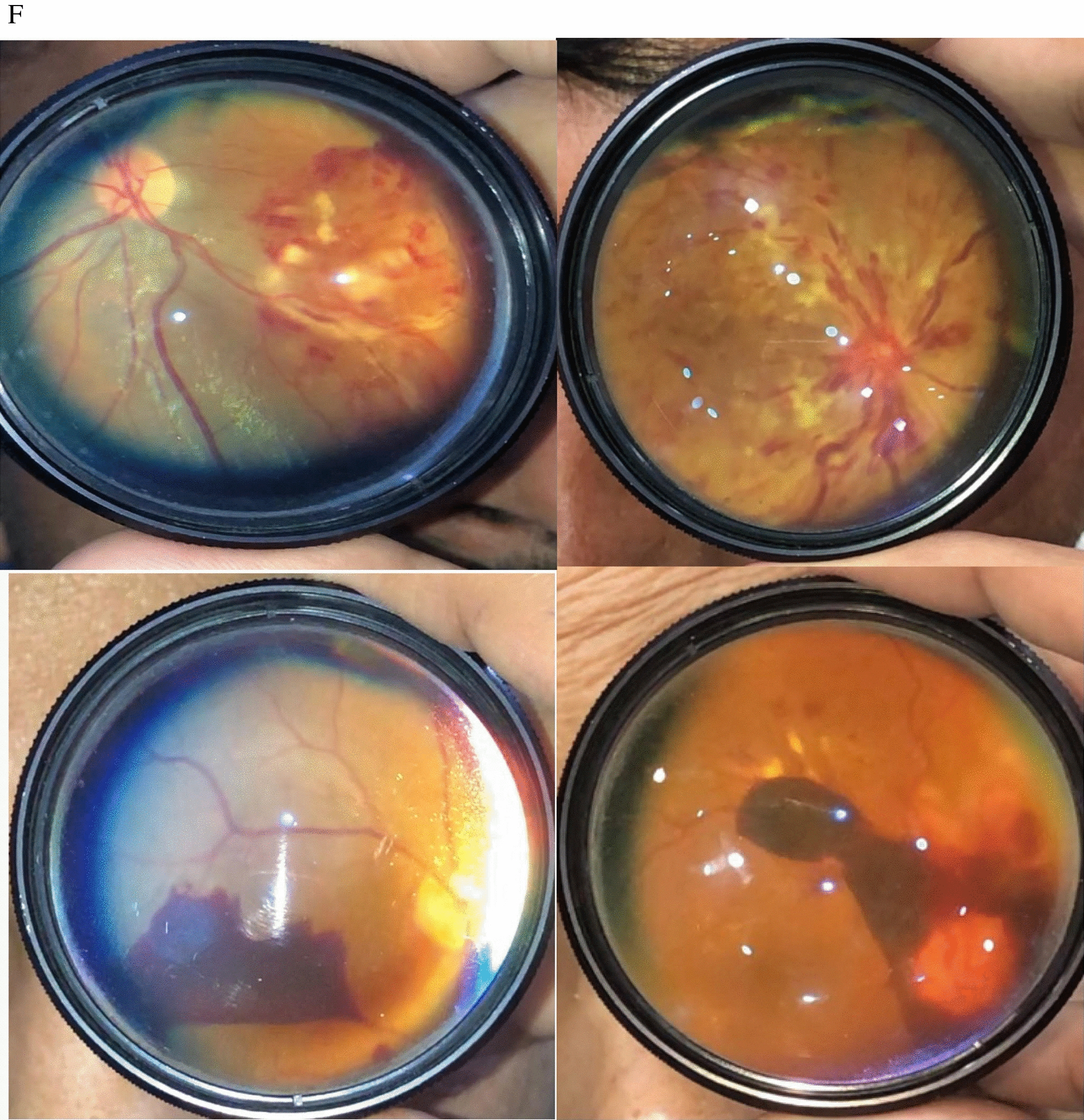
Fundus pictures showing different types of retinal hemorrhages

## Data Availability

Not applicable.

## Appendix

See Figs. 4, 5, 6, 7, 8 and 9.
